# Associations of sleep problems with asthma and allergic rhinitis among Chinese preschoolers

**DOI:** 10.1038/s41598-022-12207-3

**Published:** 2022-05-16

**Authors:** Ying Ma, Jie Tang, Yuqi Wen, Yan Hu, Jingjing Liang, Lin Jiang, Yanfei Xing, Yanyan Song

**Affiliations:** 1 Department of Child Health Care, Guangzhou Women and Children’s Medical Center, Guangzhou Medical University, 9th Jinsui Road, Tianhe District, Guangzhou, 510623 China; 2 Department of Preventive Medicine, School of Public Health, Guangzhou Medical University, Guangzhou, China

**Keywords:** Health care, Health services, Public health

## Abstract

The aim of this study was to examine the associations of sleep problems with asthma and allergic rhinitis among Chinese preschoolers. This cross-sectional survey was conducted in Guangzhou, China. Children aged 3–6 years were recruited from 32 kindergartens in 7 administrative districts. Asthma, allergic rhinitis and sleep problems were evaluated using a valid questionnaire. Binary logistic regression models were employed to estimate the odds ratios (OR) and 95% confidence intervals (CI) for the associations of asthma and allergic rhinitis with short sleep duration, late bedtime and frequent nocturnal awakening. We included 4876 preschool children in the current analysis. Of these, 182 (3.7%) diagnosed as asthma, and 511 (10.5%) diagnosed as allergic rhinitis. Frequent nocturnal awakening was associated with asthma and allergic rhinitis, with adjusted OR were 1.49 (95% CI 1.05–2.13) and 1.59 (95% CI 1.27–1.99), respectively. Subgroup analysis showed the OR for frequent nocturnal awakening with asthma was higher in girls (1.68; 95% CI 1.02–2.78) than in boys (1.35; 95% CI 0.81–2.24), but the OR for frequent nocturnal awakening with allergic rhinitis were similar in girls (1.73; 95% CI 1.15–2.30) and boys (1.57; 95% CI 1.17–2.12). No significant associations of short sleep duration and late bedtime with asthma or allergic rhinitis were identified. Our data suggested that frequent nocturnal awakening was associated with asthma and allergic rhinitis among preschoolers, and the association of frequent nocturnal awakening with asthma differed by gender. Further studies are warranted to address the causal relationship between nocturnal awakening and asthma and allergic rhinitis.

## Introduction

Allergic respiratory diseases (ARDs), primarily asthma and allergic rhinitis, are attracting important clinical and public concerns all over the world. Globally, asthma and allergic rhinitis affect 4–10% and 10–30% of the whole population^1^, respectively. Among children, the prevalence of ARDs is disparate across regions and countries, but the overall prevalence increases year by year^2–4^. ARDs not only increase health and care costs, but also reduce the life quality of sufferers^5,6^, calling for more preventive measures to minimize its detriment fundamentally.

ARDs are associated with a broad range of environmental factors and lifestyles other than genetic factors^7^, which are not fully understood. Recently, associations between sleep problems and ARDs have gained increasing attentions. Sleep problems, such as difficulty in settling to sleep, nocturnal awakening, irregular sleep patterns and short sleep duration are common in children. Approximately 25% of children experienced some forms of sleep problems during childhood^8^. To date, several studies have examined the associations between sleep problems and ARDs. However, few studies have been conducted among pre-school age children^9,10^. Previous studies have suggested that residency in urban areas, prenatal smoking and passive smoking are associated with an increased risk of wheezing and asthma^11–13^, but most available studies on sleep problems and ARDs have not adjusted for such confounders. Therefore, the associations of sleep problems with asthma and allergic rhinitis among pre-school aged children remains unclear. It is of substantial importance to identify the associations among pre-school age children, as this is a critical period for developing the physique and immune system and forming a healthy lifestyle, including developing good sleep habits^10,14^.

We hypothesized that sleep problems, including frequent nocturnal awakening, short sleep duration and late bedtime are associated with ARDs even after controlling for potential confounders.

## Methods

### Study design and participants

This study was a part of the National Survey on Physical Growth and Development of Children in nine cities of China (NSPGDC), which used the identical methodology to collect data to monitor growth and development of children aged 0–6 years^15^. The study design, organization, and implementation of the NSPGDC have been published previously^16^. Briefly, in each study city, a cluster random sampling method based on age groups (there were 22 age groups and 150–200 subjects for each sex-age subgroup) for both urban and rural areas was employed to produce a random sample^17^. Children under 3 years in a community was classified as a minimum cluster unit, and children aged 3 and above in kindergarten regarded as a unit. Exclusion criteria included temporary residents, acute illness within a month, serious diseases (such as serious congenital cardiopathy, serious hepatopathy and nephrosis, neurological disease), and malnourished and physically handicapped.

We conducted the NSPGDC in Guangzhou between July and October in 2015. Because we aimed to explore the associations of sleep problems with asthma and allergic rhinitis among preschool children, a subgroup of children aged between 3 and 6 years who participated in the NSPGDC of Guangzhou were included in the analysis. Using a random cluster sampling, we recruited 5102 children aged 3 to 6 years from 16 kindergartens in urban administrative districts (Yuexiu, Liwan and Haizhu), and 16 kindergartens in rural administrative districts (Conghua, Huadu, Panyu and Baiyun) of Guangzhou. There were 18 children declined to participate in this survey and 217 children submitted an incomplete questionnaire with missing data of > 15%. Therefore, the remaining 4867 children aged 3 to 6 years old were included for analysis. The response rate was 95.4% (4867/5102). Data were collected by local trained physicians using a structured questionnaire, which included participants’ demographic characteristics, mother’s health conditions during pregnancy, delivery mode, feeding patterns in the first 6 months, sleep problems and ARDs of the participants. Body weight and height were also measured by calibrated instruments and standard specifications, and body mass index (BMI) was calculated by dividing the weight in kilogram (kg) by the square of length in meters (m). Age and sex specific BMI z-score was calculated according to the Chinese standard^18^.

### Measurement

Asthma and allergic rhinitis were defined as having current symptoms and physician diagnosis^19^. Current symptoms of asthma and allergic rhinitis were assessed by the questions derived from the International Study of Asthma and Allergy in Children Questionnaire (ISAACQ) (“Has your baby had wheezing or whistling in the chest during the past 12 months?” “Has your baby had a problem with sneezing, or a runny, or a blocked nose when he/she did not have a cold or the flu during the past 12 months?”^2^). Information on diagnosis of asthma and allergic rhinitis were obtained by the questions of “Has your baby had ever been diagnosed with asthma by the doctor?” and “Has your baby had ever been diagnosed with allergic rhinitis by the doctor?”.

We assessed sleep duration, usual bedtime and nocturnal awakening frequency in 2 weeks preceding to the survey, using the questions derived from the Chinese version of the Children’s Sleep Habits Questionnaire (CSHQ)^20^. Sleep duration was assessed based on the following question “What is your child’s usual amount of sleep each day (combining nighttime sleep and naps)”. According to the National Sleep Foundation’s recommendation, preschoolers (3–6 years) who slept for less than 10 h were defined as short sleep duration^21^. Bedtime was assessed by the question “What is your child’s usual bedtime?”. As the 75 percentiles of bedtime among 3 to 6 aged children was 22:00, the bedtime was classified into 2 groups: at or before 22:00 and after 22:00, and bedtime later than 22:00 was considered as late bedtime. The nocturnal awakening frequency was assessed by the question “What is your child’s number of wake-up times during the night?”, and classified into 2 groups: none or seldom, and once or more per night. According to the previous study by the National Sleep Foundation^22^, children who wakened once or more per night among preschoolers (3–6 years) were defined as frequent nocturnal awakening.

Previous studies suggested a broad range of demographic characteristics and environmental factors that were associated with asthma and allergic rhinitis^9,10,23,24^. Therefore, we adjusted for these potential confounders, which was distributed differently according to the allergic disease in our analysis, including resident area, age, gender, mother’s education, BMI z-score of children, delivery mode, birth weight, maternal tobacco exposure during pregnancy, and feeding patterns in the first 6 months.

### Ethics declarations

The study was approved by the Ethical Committee for Biomedical Research in Guangzhou Women and Children’s Medical Center, and was conducted in accordance with Helsinki Declaration and Ethical Guidelines for Research Involving Human Participants. A written informed consent was obtained from all the participants’ parents before starting of the survey.

### Statistical analysis

Mean and standard deviation were reported for continuous variables. Frequencies and percentages were reported for categorical variables. T tests and Chi-square tests were used for comparing continuous and categorical variables, respectively. Binary logistic regression models were employed to estimate the odds ratios (OR) and 95% confidence intervals (CI) for the associations of asthma and allergic rhinitis with short sleep duration, late bedtime and frequent nocturnal awakening, respectively. In each logistic regression model, three models were fitted.

In model 1, we estimated the crude ORs. In model 2, we adjusted for demographic characteristics, included region (urban/rural), gender (boys/girls), age, mother’s educational level (college or above/senior high school/junior high school or below) and BMI z-score (continuous data). In model 3, we additionally adjusted for delivery mode (vaginal delivery/cesarean delivery), birth weight (< 2500 g/2500–3900 g/≥ 4000 g), maternal tobacco exposure (smoking or passive smoking) during pregnancy, feeding patterns in the first 6 months of the children (breastfeeding/artificial feeding/mixed feeding), passive smoking (yes/no).

We further conducted subgroup analysis to examine the gender influence on the associations of asthma/allergic rhinitis with frequent nocturnal awakening where significant associations were found. In subgroup analysis, we adjusted for the covariates as we did in model 3. Missing data of continuous covariates was inputted based on means and categorical covariates was inputted by the median. Significance level was set at *P* < 0.05. To reduce the potential type I error due to multiple comparisons in subgroup analyses, significance level was adjusted using the Bonferroni method, and all tests were 2-sided^25^. Statistical analyses were conducted using SPSS Statistics, version 25.0 (IBM Corp).

## Results

### Participants’ characteristics

We included 4867 children in the current analysis. Of them, 2518 (51.7%) were boys and 2383 (49.0%) were from the urban areas. The mean age of the participants was 4.28 ± 1.05 years. Other characteristics of the participants were summarized in Table 1. Overall, 182 (3.7%) participants were diagnosed as asthma. Compared with the participants without asthma, participants with asthma were more likely to be living in urban areas, with low birth weight and exposed to tobacco during pregnancy. There were 511 (10.5%) participants diagnosed as allergic rhinitis. Compared with participants without allergic rhinitis, those with allergic rhinitis were more likely to be living in urban areas, boys, cesarean delivered, less breast-feeding and having higher maternal educational level (see Table 1).

**Table 1:** The characteristics of the participants.

Characteristics		Total (n = 4867)	Asthma		Allergic rhinitis	
			Yes (n = 182)	No (n = 4685)	Yes (n = 511)	No (n = 4356)
Region	Urban	2383 (49.0)	103 (56.6)*	2280 (48.7)	283 (55.4)*	2100 (48.2)
	Rural	2484 (51.0)	79 (43.4)	2405 (51.3)	228 (44.6)	2256 (51.8)
Gender	Boys	2518 (51.7)	93 (51.1)	2425 (51.8)	300 (58.7)*	2218 (50.9)
	Girls	2349 (48.3)	89 (48.9)	2260 (48.2)	211 (41.3)	2138 (49.1)
Age	3 years	1448 (29.8)	58 (31.9)	1390 (29.7)	124 (24.3)*	1324 (30.4)
	4 years	1381 (28.4)	56 (30.8)	1325 (28.3)	144 (28.2)	1237 (28.4)
	5 years	1273 (26.2)	49 (26.9)	1224 (26.1)	161 (31.5)	1112 (25.5)
	6 years	765 (15.7)	19 (10.4)	746 (15.9)	82 (16.0)	683 (15.7)
Ethnicity	Han	4767 (97.9)	178 (97.8)	4589 (98.0)	503 (98.4)	4264 (97.9)
	Others	100 (2.1)	4 (2.2)	96 (2.0)	8 (1.6)	92 (2.1)
Mother’s educational level	College or above	3205 (65.9)	133 (73.1)	3072 (65.6)	374 (73.2)*	2831 (65.0)
	Senior high school	1063 (21.8)	29 (15.9)	1034 (22.1)	83 (16.2)	980 (22.5)
	Junior high school or below	599 (12.3)	20 (11.0)	579 (12.4)	54 (10.6)	545 (12.5)
Mother’s occupation	Famers	36 (0.7)	0 (0)	36 (0.8)	6 (1.2)	30 (0.7)
	Workers	1467 (30.1)	65 (35.7)	1402 (329.9)	151 (29.5)	1316 (30.2)
	Servicer	977 (20.1)	33 (18.1)	944 (20.1)	106 (20.7)	871 (20.0)
	Others	2387 (49.0)	84 (46.2)	2303 (49.2)	248 (48.5)	2139 (49.1)
Family annual income (RMB)	< 50,000	567 (11.6)	15 (8.2)	5523 (11.8)	58 (11.4)	509 (11.7)
	~ 100,000	1262 (25.9)	46 (25.3)	1216 (26.0)	125 (24.5)	1137 (26.1)
	~ 300,000	2292 (47.1)	89 (48.9)	2203 (47.0)	263 (51.5)	2029 (46.6)
	> 300,000	746 (15.3)	32 (17.6)	714 (15.2)	65 (12.7)	681 (15.6)
Delivery methods	Vaginal delivery	2587 (53.2)	94 (51.6)	2493 (53.2)	249 (48.7)*	2338 (53.7)
	Caesarean delivery	2280 (46.8)	88 (48.4)	2192 (46.8)	262 (51.3)	2018 (46.3)
Preterm birth	Yes	134 (2.8)	9 (4.9)	125 (2.7)	19 (3.7)	115 (2.6)
	No	4733 (97.2)	173 (95.1)	4560 (97.3)	492 (96.3)	4241 (97.4)
Birth weight (g)	< 2500	74 (1.5)	5 (2.7) *	69 (1.5)	9 (1.8)	65 (1.5)
	2500–3999	4610 (94.7)	173 (95.1)	4437 (94.7)	489 (95.7)	4121 (94.6)
	≥ 4000	183 (3.8)	4 (2.2)	179 (3.8)	13 (2.5)	170 (3.9)
Feeding patterns in the first 6 months	Breastfeeding	1585 (32.6)	45 (24.7)	1540 (32.9)	37 (26.8)*	1448 (33.2)
	Artificial feeding	873 (17.9)	33 (18.17)	840 (17.9)	101 (19.8)	772 (17.7)
	Mixed feeding	2409 (49.5)	104 (57.1)	2305 (49.2)	273 (53.4)	2136 (49.0)
Maternal tobacco exposure during pregnancy	Yes	1694 (34.8)	76 (41.8)*	1618 (34.5)	194 (38.0)	1500 (34.4)
	No	3173 (65.2)	106 (58.2)	3067 (65.5)	317 (62.0)	2856 (5.6)
Passive smoking of children	Yes	1969 (40.5)	82 (45.1)	1887 (460.3)	215 (42.1)	1754 (40.3)
	No	2898 (59.5)	100 (54.9)	2798 (59.7)	296 (57.9)	2602 (59.7)
Children’s BMI z-score		0.09 ± 0.98	0.14 ± 0.97	0.09 ± 0.98	0.03 ± 0.92	0.10 ± 0.99

*P < 0.05.

### Association between sleep problems and asthma

Of the 4867 children included, 292 (6.0%) had a short sleep duration. Among the children who diagnosed as asthma, 10 (5.5%) had a short sleep duration; while among the children without asthma, 282 (6.0%) had a short sleep duration. No significant association between short sleep duration and asthma was found (Table 2). There were 976 (20.1%) participants went to sleep after 22:00. Among the children with asthma, 41 (22.5%) had late bedtime, while in the group without asthma diagnosis, 935 (20.0%) went to sleep lately. There was also no significant association between late bedtime and asthma (Table 2). There were 816 (16.8%) participants who had frequent nocturnal awakening, of whom 42 (23.1%) participants diagnosed as asthma and 774 (16.5%) participants did not have an asthma diagnosis. A significant association between frequent nocturnal awakening and asthma was found (Table 2).

**Table 2:** Frequency and proportion of sleep problems by asthma and allergic rhinitis.

Sleep problems	Total (N = 4867)	Asthma (n/%)		χ^2^	*P*	Allergic rhinitis (n/%)		χ^2^	*P*
		Yes (N = 182)	No (N = 4685)			Yes (N = 511)	No (N = 4356)		
**Short sleep duration**									
No	4575 (94.0)	172 (94.5)	4403 (94.0)	0.09	0.77	470 (92.0)	4105 (94.2)	4.15	0.04
Yes	292 (6.0)	10 (5.5)	282 (6.0)			41 (8.0)	251 (5.8)		
**Late bedtime**									
No	3891 (19.9)	141 (77.5)	3750 (80.0)	0.72	0.40	391 (76.5)	3500 (80.3)	4.19	0.04
Yes	976 (20.1)	41 (22.5)	935 (20.0)			120 (23.5)	856 (19.7)		
**Frequent nocturnal awakening**									
No	4051 (83.2)	140 (76.9)	3911 (83.5)	5.40	0.02	395 (77.3)	3656 (83.9)	14.41	< 0.001
Yes	816 (16.8)	42 (23.1)	774 (16.5)			116 (22.7)	700 (16.1)		

The unadjusted and adjusted ORs for the association between an asthma diagnosis and sleep problems were showed in Table 3. From model 1 to model 3, the ORs did not change substantially. In the fully adjusted models, a significant association was found between frequent nocturnal awakening and asthma, the OR was 1.49 (95% CI 1.05–2.13). Further subgroup analysis showed a significant association between frequent nocturnal awakening and asthma was found among girls but not among boys (Table 4).

**Table 3:** Association of sleep problems with asthma or allergic rhinitis among per-school children.

	Model 1^a^	*P*	Model 2^b^	*P*	Model 3^c^	*P*
	OR (95% CI)		OR (95% CI)		OR (95% CI)	
**Sleep problems and asthma**						
Short sleep duration						
No	1 (Ref)		1 (Ref)		1 (Ref)	
Yes	0.91 (0.47–1.74)	0.770	0.98 (0.50–1.89)	0.940	0.95 (0.49–1.84)	0.867
Late bedtime						
No	1 (Ref)		1 (Ref)		1 (Ref)	
Yes	1.17 (0.82–1.66)	0.396	1.12 (0.78–1.60)	0.535	1.11 (0.78–1.58)	0.574
Frequent nocturnal awakening						
No	1 (Ref)		1 (Ref)		1 (Ref)	
Yes	1.52 (1.07–2.16)	0.021	1.50 (1.05–2.13)	0.026	1.49 (1.05–2.13)	0.028
**Sleep problems and allergic rhinitis**						
Short sleep duration						
No	1 (Ref)		1 (Ref)		1 (Ref)	
Yes	1.43 (1.01–2.01)	0.043	1.26 (0.89–1.80)	0.197	1.23 (0.86–1.76)	0.247
Late bedtime						
No	1 (Ref)		1 (Ref)		1 (Ref)	
Yes	1.26 (1.01–1.56)	0.041	1.25 (1.01–1.56)	0.045	1.24 (0.99–1.54)	0.056
Frequent nocturnal awakening						
No	1 (Ref)		1 (Ref)		1 (Ref)	
Yes	1.53 (1.23–1.91)	< 0.001	1.59 (1.27–1.99)	< 0.001	1.59 (1.27–1.99)	< 0.001

^a^Model 1: Unadjusted.

^b^Model 2: Adjusted for region, gender, age, mother’s educational level, and BMI z-score.

^c^Model 3: Additionally adjusted for delivery mode, birth weight, and maternal tobacco exposure during pregnancy, feeding pattern before 6 months.

**Table 4:** Association between frequent nocturnal awakening and asthma according to gender.

Frequent nocturnal awakening	No. of asthma	Model a	*P*	Model b	*P*
		OR (95% CI)		OR (95% CI)	
**Boy**					
No (N = 2086)	73 (3.5)	1 (Ref)		1 (Ref)	
Yes (N = 432)	20 (4.6)	1.34 (0.81–2.22)	0.259	1.35 (0.81–2.24)	0.253
**Girl**					
No (N = 1965)	67 (3.4)	1 (Ref)		1 (Ref)	
Yes (N = 384)	22 (5.7)	1.72 (1.05–2.82)	0.031	1.68 (1.02–2.78)	0.042

Model a: Unadjusted.

Model b: Adjusted for region, age, mother’s educational level, BMI z-score, delivery mode, birth weight, maternal tobacco exposure during pregnancy, feeding pattern before 6 months.

### Association between sleep problems and allergic rhinitis

Among participants diagnosed with allergic rhinitis, 41 (8.0%) had short sleep duration, 120 (23.1%) had late bedtime, and 116 (22.7%) had frequent nocturnal awakening; while among participants without allergic rhinitis diagnosis, 251 (5.8%) had short sleep duration, 856 (19.7%) had a late bedtime, and 700 (16.1%) had frequent nocturnal awakening, respectively. Significant associations between short sleep duration, late bedtime, frequent nocturnal awakening and allergic rhinitis were found (Table 2 and Model 1 in Table 3). However, significant association was only found between frequent nocturnal awakening and allergic rhinitis after adjusted for potential confounders (Table 3). In the fully adjusted model (Model 3), the adjusted OR for the association of allergic rhinitis with frequent nocturnal awakening was 1.59 (95% CI 1.27–1.99). And the association between frequent nocturnal awakening and allergic rhinitis was found both in boys and girls (Table 5).

**Table 5:** Association between frequent nocturnal awakening and allergic rhinitis according to gender.

Frequent nocturnal awakening	No. of allergic rhinitis	Model a	*P*	Model b	*P*
		OR (95% CI)		OR (95% CI)	
**Boy**					
No (N = 2086)	232 (11.1)	1 (Ref)		1 (Ref)	
Yes (N = 432)	68 (15.7)	1.49 (1.11–2.00)	0.007	1.57 (1.17–2.12)	0.003
**Girl**					
No (N = 1965)	163 (8.3)	1 (Ref)		1 (Ref)	
Yes (N = 384)	48 (12.5)	1.58 (1.12–2.22)	0.009	1.73 (1.15–2.30)	0.006

Model a: Unadjusted.

Model b: Adjusted for region, age, mother’s educational level, BMI z-score, delivery mode, birth weight, maternal tobacco exposure during pregnancy, feeding pattern before 6 months.

## Discussion

In this study, we used a representative citywide survey data, which was part of the NSPGDC, to investigate the associations of sleep problems with asthma and allergic rhinitis among children aged 3–6 years in Guangzhou. The results revealed the prevalence of asthma and allergic rhinitis among preschool children were 3.7%, and 10.5% respectively. The findings also suggested that frequent nocturnal awakening but not short sleep duration and late bedtime, was significantly associated with asthma and allergic rhinitis. However, these associations of frequent nocturnal awakening and asthma differed by gender.

The prevalence of asthma and allergic rhinitis among preschool children was consistent with previous studies. For example, Deng et al.’s research in Beijing among preschool children revealed the prevalence of doctor-diagnosed childhood asthma was 2.8%^26^. Similarly, Pereira et al. conducted a study in Portuguese children aged 3–5 years and the prevalence of physician-diagnosed asthma was 4.6%, and 11.8% for the physician-diagnosed rhinitis^27^. Deng et al.’s research in Changsha in China exhibited the prevalence of ever doctor-diagnosed allergic rhinitis was 7.3%^28^. Bloom et al.’s research in UK showed the prevalence of preschool wheeze requiring attendance to a physician in 2017 was 7.7%, but only one fifth of the preschool children with wheeze received an asthma diagnosis^29^. However, in six representative cities in China, the average prevalence of asthma and allergic rhinitis among preschool children were higher than ours, with 8.0% for asthma and 16.6% for allergic rhinitis, respectively^30^. Previously, regional difference in the prevalence of asthma and allergic rhinitis were found^30^, and our result was consisted with the Urumqi’s prevalence, showing prevalence of 3.5% for asthma and 10.9% for allergic rhinitis. The discrepancies might be related to different characteristics of study participants (residence, ethnicity), apart from the different measurements for asthma and allergic rhinitis. Besides, the under-diagnosis although with symptom underestimated the prevalence of asthma and allergic rhinitis^29^.

Our findings suggested that frequent nocturnal awakening was associated with asthma (OR 1.49, 1.05–2.13) and allergic rhinitis (OR 1.59, 1.27–1.99), which was similar to previous studies that investigated the associations between sleep problems and allergic diseases among children and adolescent^9,10,31^. Wang et al. conducted a study to examine the associations of sleep disorders with the risk of wheeze and allergic rhinitis among 566 Chinese toddlers and found that having more than 2 times of nocturnal awakening per night was associated with a higher risk of wheeze (OR 6.16, 1.28–29.74)^9^. Kozyrskyj et al. analyzed the conditions of 2398 children from on a community-based birth cohort in Australia and found that persistent nocturnal awakening before 3 years of age was associated with an increased risk of non-atopic asthma at age 6 (OR 1.87, 1.08–3.25), after adjusting for other risk factors of asthma, including co-sleeping, wheeze and family stress^10^. Jernelöv et al. presented data from a longitudinal study showing that children overtired at the age of 8 years increased the risk of rhinitis symptoms at age 13^31^.

The associations between frequent nocturnal awakening and asthma/allergic rhinitis could be explained from two aspects. On one hand, the nocturnal awakening may be consequence of asthma or allergic rhinitis, as the airway inflammation and congestion or the nasal obstruction, may cause them to wake up frequently. There is evidence showing high prevalence of nocturnal awakening caused by asthma or allergic rhinitis among children^32–34^. On the other hand, the frequent nocturnal awakening may increase risk of asthma or allergic rhinitis, which was demonstrated in a longitudinal study^10^. The possible biological mechanism was as follow: first, frequent nocturnal awakening may increase the levels of pro-inflammatory cytokines and decrease the immunologic tolerance to allergen^9,10^, shifting the balance between Th1 and Th2 cytokines towards an allergy related (Th2) pattern^31^, which are known factors contributing to allergic diseases such as asthma and allergic rhinitis. Second, frequent nocturnal awakening could disrupt the regulation of the hypothalamic–pituitary–adrenal (HPA) axis^35,36^ and the circadian rhythms of melatonin^37,38^, which showed blunted cortisol awakening response and lower cortisol levels and a decline in the level of melatonin^35,36^, and increase the risk of asthma and rhinitis^39–42^. In summary, the association between frequent nocturnal awakening and asthma/allergic rhinitis might work in both directions. However, as our study was a cross-sectional study, we cannot ascribe causality of the association of frequent nocturnal awakening and asthma/allergic rhinitis, further research to address the causal relationship between sleep problems and allergic diseases seems warranted.

In this study, we also found that the associations of frequent nocturnal awakening with asthma differed by gender, showing only associations in girls. Few studies have detected the gender difference of association between sleep problems and allergic disease among preschooler in community population, and our finding was consistent with the studies conducted in asthma patients. For example, Strunk et al. showed that night awakening caused by asthma was marginally less for males than females^30^. Goldstein et al. also found that the association of asthma with sleep-disordered breathing was only significant in girls but not in boys^43^. The gender differences may be driven by hormonal effect on school-age children^44^. However, it is noticed that the sex difference of sex hormonal will not be significant, until the onset of puberty^45^. Therefore, whether the explanation can be applied to preschoolers need further studies to verify.

### Strengths and limitations

For this citywide cross-sectional study, we recruited 4867 representative participants from rural and urban in Guangzhou by using a well-designed protocol, which makes our results more generalized. In the analysis we have adjusted for several important confounders, such as region and maternal tobacco exposures during pregnancy, which were not adjusted in previous studies, making our results more robust. Additionally, there were few studies that explored the associations of sleep problems with asthma/ allergic rhinitis among Chinese preschool children in a large sample size, and there were no studies that explored the gender difference of the associations in community population.

There were several limitations in our study. First, asthma and allergic rhinitis were not determined by objective measures, rather than parents’ reported symptom from ISAAC and previous diagnosis. However, although weak, the objective measurement is highly recognized for its reliability and validity and was used in previous studies^10,31,46^. Second, sleep problems were assessed through questionnaires based on the parents’ reports, instead of objective measurement, thus recall bias may exist. On balance, previous studies have demonstrated that information regarding sleep garnered from parents is likely to be reliable^47^. Third, although we have adjusted for various potential confounders, we did not adjust for family allergy history of the parents or mother’s stress during pregnancy, which are risk factors for allergenic diseases^10^. Fourth, this study was a cross-sectional study, which deduced a weak association in the exploration of the causal relationship of sleep problems with asthma and allergic rhinitis. Future prospective research studies are needed to validate these findings, since asthma and allergic rhinitis are of significant clinical and public concerns.

Sleep problems including shortened sleep duration, late bedtime and frequent nocturnal awakening are common among children, which have a broad impact on children’s development and physical health. In the present study, we used data from the NSPGDC to investigate the associations of sleep problems with asthma and allergic rhinitis among Chinese preschool children and found that frequent nocturnal awakening was associated with asthma and allergic rhinitis, and the association of frequent nocturnal awakening with asthma differed by gender. The results suggested that pediatricians should consider evaluating sleep problems when appraising the consequence or modifiable factors of asthma and allergic rhinitis and developing good sleeping habits may be beneficial for asthma and allergic rhinitis.

## Conclusions

Findings from this well representative study suggested that frequent nocturnal awakening was associated with asthma and allergic rhinitis, and the association of frequent nocturnal awakening with asthma differed by gender. Further studies are warranted to address the causal relationship between nocturnal awakening and asthma and allergic rhinitis.

## Data Availability

Data and material of this study could be available through contacting with the corresponding authors. Data of NSPGDC can’t be available because its’ intellectual property belongs to Guangzhou Health and Family Planning Commission.

## Abbreviations

ARDs: Allergic respiratory diseases
BMI: Body mass index
CI: Confidence intervals
NSPGDC: National Survey on Physical Growth and Development of Children in nine cities of China
OR: Odds ratio
